# Grass-like plants release general volatile cues attractive for gravid *Anopheles gambiae* sensu stricto mosquitoes

**DOI:** 10.1186/s13071-021-04939-4

**Published:** 2021-10-27

**Authors:** Getachew E. Bokore, Linus Svenberg, Richard Tamre, Patrick Onyango, Tullu Bukhari, Åsa Emmer, Ulrike Fillinger

**Affiliations:** 1 International Centre of Insect Physiology and Ecology, P.O. Box 30772–00100, Nairobi, Kenya; 2 School of Physical and Biological Sciences, Department of Zoology, Maseno University, Maseno, Kenya; 3 Ethiopian Public Health Institute, P. O. Box 1242, Addis Ababa, Ethiopia; 4 Analytical Chemistry, Div. of Applied Physical Chemistry, Dept. of Chemistry, KTH Royal Institute of Technology, Stockholm, Sweden

**Keywords:** Attractants, Gravid mosquitoes, Malaria, Graminoid plants, Olfactometer, Plant volatiles, Semi-field, Vector control, Attract-and-kill

## Abstract

**Background:**

Understanding the ecology and behaviour of disease vectors, including the olfactory cues used to orient and select hosts and egg-laying sites, are essential for the development of novel, insecticide-free control tools. Selected graminoid plants have been shown to release volatile chemicals attracting malaria vectors; however, whether the attraction is selective to individual plants or more general across genera and families is still unclear.

**Methods:**

To contribute to the current evidence, we implemented bioassays in two-port airflow olfactometers and in large field cages with four live graminoid plant species commonly found associated with malaria vector breeding sites in western Kenya: *Cyperus rotundus* and *C. exaltatus* of the Cyperaceae family, and *Panicum repens* and *Cynodon dactylon* of the Poaceae family. Additionally, we tested one Poaceae species, *Cenchrus setaceus*, not usually associated with water. The volatile compounds released in the headspace of the plants were identified using gas chromatography/mass spectrometry.

**Results:**

All five plants attracted gravid vectors, with the odds of a mosquito orienting towards the choice-chamber with the plant in an olfactometer being 2–5 times higher than when no plant was present. This attraction was maintained when tested with free-flying mosquitoes over a longer distance in large field cages, though at lower strength, with the odds of attracting a female 1.5–2.5 times higher when live plants were present than when only water was present in the trap. *Cyperus rotundus,* previously implicated in connection with an oviposition attractant, consistently elicited the strongest response from gravid vectors. Volatiles regularly detected were limonene, β-pinene, β-elemene and β-caryophyllene, among other common plant compounds previously described in association with odour-orientation of gravid and unfed malaria vectors.

**Conclusions:**

The present study confirms that gravid *Anopheles gambiae* sensu stricto use chemical cues released from graminoid plants to orientate. These cues are released from a variety of graminoid plant species in both the Cyperaceae and Poaceae family. Given the general nature of these cues, it appears unlikely that they are exclusively used for the location of suitable oviposition sites. The utilization of these chemical cues for attract-and-kill trapping strategies must be explored under natural conditions to investigate their efficiency when in competition with complex interacting natural cues.

**Graphical abstract:**

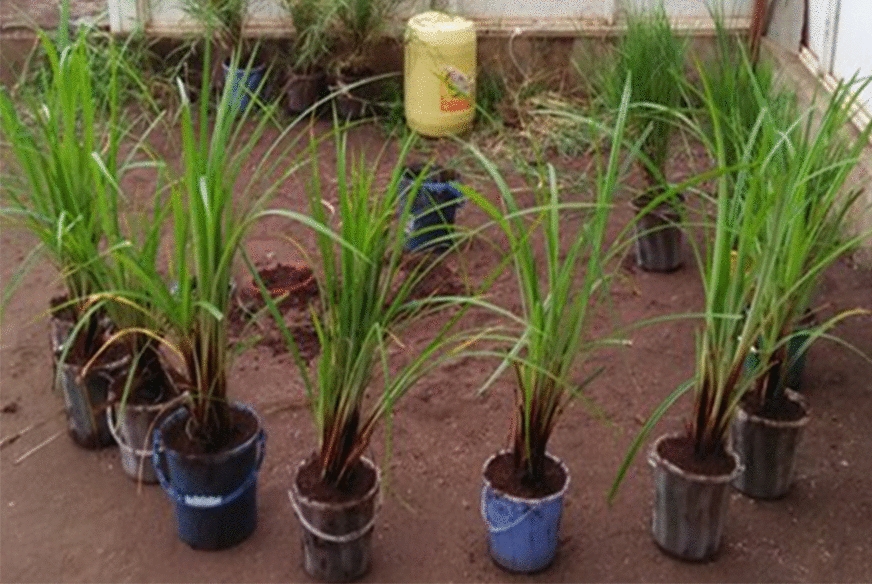

**Supplementary Information:**

The online version contains supplementary material available at 10.1186/s13071-021-04939-4.

## Background

Mosquitoes use visual, olfactory, and tactile cues for survival and reproduction in a complex environment [1]. Understanding the ecology and behaviour of disease vectors, including the olfactory cues used to orient and select hosts and egg-laying sites, is essential for the development of novel, insecticide-free control tools [2–4]. The outdoor behaviour of Afro-tropical malaria vectors has gained increased attention over the past decade, after a realization that interventions targeted at the indoor environment alone will not be sufficient to eliminate malaria from most locations in sub-Saharan Africa [5–7]. Tools that complement long-lasting insecticidal nets (LLINs) and indoor residual sprays (IRS) need to combat physiological insecticide resistance and address behavioural insecticide avoidance such as outdoor feeding and resting [3, 8, 9]. For their reproductive success, malaria vector mosquitoes depend on finding and selecting a suitable aquatic habitat for egg-laying and development of their immature stages [10–12]. The need for an aquatic habitat unites all female vectors irrespective of their feeding and resting behaviour, and degree of resistance to insecticides. This provides an opportunity to target this physiological stage for control. Studying the chemical ecology of the egg-laying behaviour of gravid malaria vectors will increase our knowledge on the sources, role and importance of volatile organic compounds (VOCs) regulating the communications between mosquitoes and their environment and might consequently facilitate the development of novel vector control and surveillance tools [13]. A range of sources for putative, attractive or repellent, chemical oviposition cues have been implicated in the literature for malaria vector mosquitoes, including from conspecific immature stages, predators, competitors, microbes, water, soil, plants and plant-based infusions [14–23].

Emergent vegetation, including graminoid plants, are frequently associated with high numbers of *Anopheles* larvae in aquatic habitats in ecological larval habitat risk factor surveys [24–29]. The vegetation might provide coverage from predators [30, 31], support microbes that contribute indirectly or directly to nutrition of the mosquito immature stages [32–35], and consequently improve survival. It is therefore plausible to hypothesize that cues from habitat-associated vegetation are used by gravid females for location of suitable breeding sites. It is well documented that plants emit VOCs that play important roles in the plants’ interactions with their environments, including insects [36–39]. Graminoid plants found in and around natural aquatic habitats have been suggested to be associated with oviposition site selection of gravid malaria vectors [17, 22, 40–42].

For example, studies have shown that gravid malaria vectors are attracted to headspace volatiles released from wetland rice plants (*Oryza* sp. [22]) and to volatiles released from pollen of maize (*Zea mays* [43]) and sugar cane (*Saccharum officinarum* [44]). The authors of that work suggest that mosquitoes have selectively adapted to habitats dominated by agricultural grasses of the Poaceae family which in turn would suggest that these grasses release a unique odour profile that separates them from native (non-agricultural) grasses.

On the other hand, the grass-like sedges in the Cyperaceae family are frequently indicators of wetlands [45, 46] and have been associated with productive *Anopheles* breeding sites in a multitude of studies [28, 40, 47]. Cedrol, a sesquiterpene alcohol, was identified from the headspace of aqueous infusions that were made from soil and rhizomes taken from a productive *Anopheles* habitat, that was densely vegetated by the sedge, *Cyperus rotundus*. The infusion as well as water treated with synthetic cedrol attracted *An. gambiae* and *An. arabiensis* in laboratory, semi-field and field experiments [17, 21]. Plant-based chemical compounds might either be released from roots and submerged plant parts into the water [36, 48] of the potential oviposition site or might be released into the air from the emergent parts of the plant [36, 49]. Cedrol has been identified directly from rhizome extracts of sedges [50] as well as from associated microbes [41].

It is against this background that we set out to contribute to the current knowledge base by further investigating native graminoid plant species from the Cyperaceae and Poaceae families for their potential to attract gravid malaria vectors with the volatiles they release when present as intact plants. The four selected test plant species dominate natural aquatic habitats around the shores of Lake Victoria in western Kenya [40]. For comparison, an ornamental dry-land grass, of the Poaceae family, usually not associated with malaria vector breeding sites was included in the study. The overall aim of this work was to investigate whether chemical cues released from graminoid plants result in species- or family-specific volatile profiles and selective responses from gravid *An. gambiae*, or whether the chemical cues are of a more general nature.

## Methods

### Study site

All experiments and plant volatile collections were conducted under ambient climate conditions at the International Centre of Insect Physiology and Ecology (icipe), Thomas Odhiambo Campus (TOC), Mbita (00° 26′ 06.19′′ S; 34° 12′ 53.13′′ E; 1137 m above sea level), western Kenya. The area is characterized by an equatorial tropical climate with daily average minimum and maximum temperatures ranging from 16 °C to 28 °C. The chemical analyses of the volatile samples were done at laboratories at KTH Royal Institute of Technology in Stockholm, Sweden.

### Gravid mosquito preparation

*Anopheles gambiae* sensu stricto (s.s.) Mbita strain insectary-reared mosquitoes were used for all experiments. Mosquitoes were reared under ambient conditions following the protocol described by Okal et al. [51]. Adult mosquitoes were held in 30 × 30 × 30 cm netting-covered cages at 25–28 °C and 68–75% relative humidity in a 12 h:12 h light/dark photoperiod. Equal numbers of 2–3-day-old adult female and male mosquitoes were transferred into a clean cage and starved for 6 h starting at 13:00 before they were allowed to feed on a human arm at 19:00 for 15 min. Blood feeding was repeated the next day at 19:00 using the same procedure. After each blood meal, the mosquitoes were provided with 6% glucose solution ad libitum. A wet towel was placed on top of the cages to provide additional humidity. After the second blood meal, the mosquitoes were kept for another 2 days with access to glucose solution. On the third day, gravid females were selected and used in bioassays.

### Preparation of test substrates

Four graminoid plant species, naturally occurring frequently in malaria vector breeding sites in western Kenya [40], namely the grass-like sedges (Cyperaceae), *C. rotundus* (nut grass), and *C. exaltatus* (giant sedge), as well as the true grasses (Poaceae), *Panicum repens* (torpedo grass) and *Cynodon dactylon* (Bermuda grass) were collected from wetlands along the shores of Lake Victoria, around Mbita and Rusinga towns, western Kenya. The plants were carefully uprooted and the plants with soil transported to *icipe*-TOC for bioassays in olfactometers and large field cages, and for volatile collections. A drought-tolerant grass, not native to wetlands and frequently used as ornamental grass in gardens, *Cenchrus setaceus* (purple fountain grass; Poaceae) was obtained from plant nurseries in Kisumu town and maintained at *icipe*-TOC. The plants were used only in their non-flowering stage (roots, stems and leaves only) in order to standardize the experiments (flowering plants likely release different odours than non-flowering) and be in the position to have sufficient plant material at any time. In preparation for bioassays, the plants were washed thoroughly using lake water to remove the soil. Fresh plant samples were used for every round of bioassays. A bunch of several individual plants, weighing approximately 350 g, was used for every replicate bioassay.

Soil collected from the habitat where *C. rotundus* was uprooted was used for a preliminary bioassay. The soil was taken from the upper 10 cm of the habitat and plant material sieved out before use. For each replicate bioassay, 4 kg of fresh soil was used.

Water was used in all bioassays (4 l per test substrate), acknowledging that water vapour is a major oviposition attractant [20]. The water originated from Lake Victoria and sediments allowed to settle before the clear supernatant was used for experiments.

A hay-infusion previously shown to be repellent for gravid *An. gambiae* [21] was prepared for the initial calibration of the olfactometer bioassays. The infusion was prepared by mixing 24 l of lake water and 90 g of hay in a bucket and kept in a dark place with the temperature ranging from 18 °C to 29 °C for 3 days before use for the bioassays. Before use, buckets were thoroughly cleaned with odourless soap and allowed to dry under the sun.

### Two-port airflow olfactometer bioassays

**Fig. 1:**
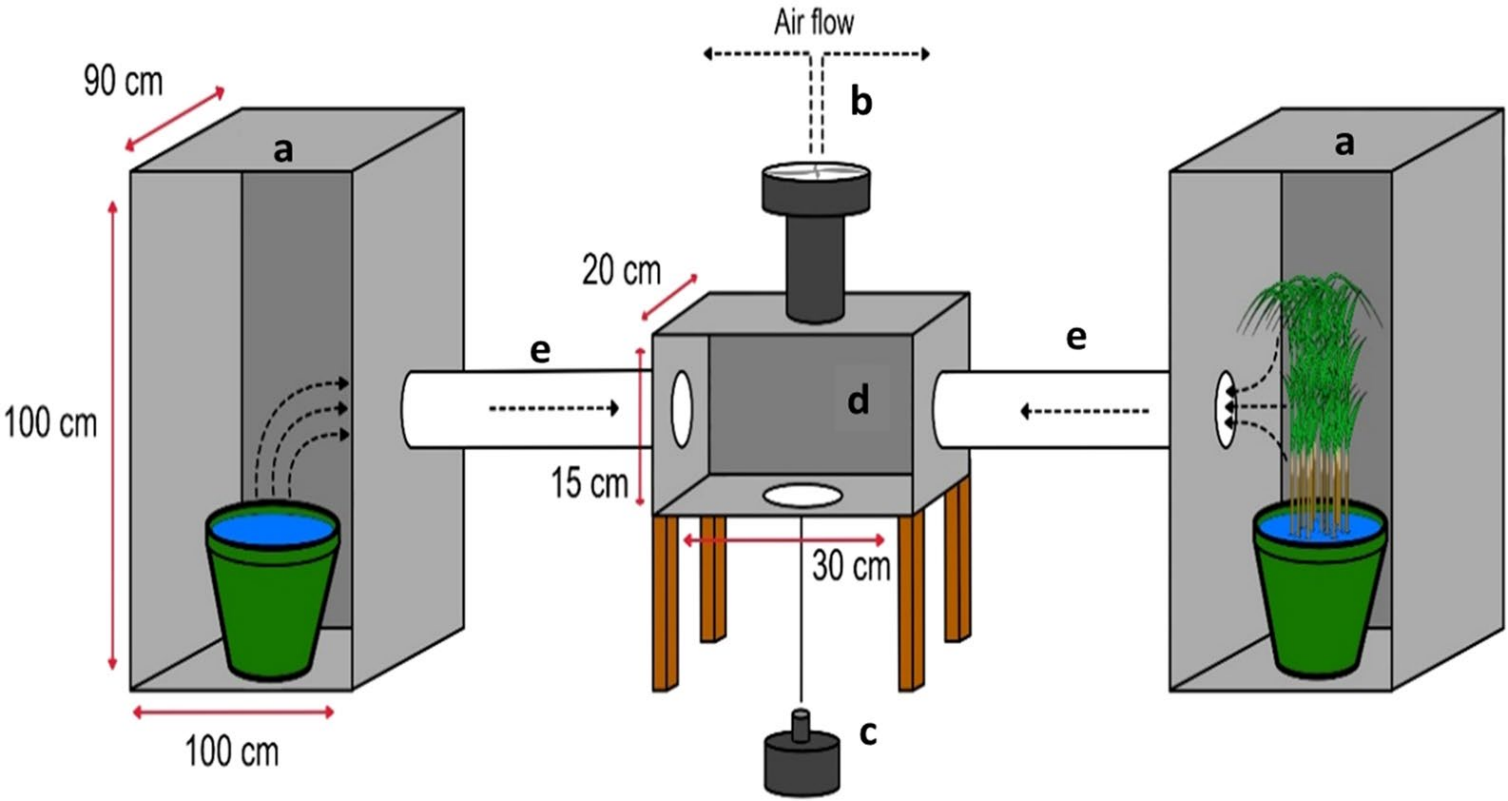
The olfactometer bioassay experimental setup. The substrates were placed in the two large (1 × 0.9 × 1 m) chambers (**a**) from which a 12-V electric fan (**b**) drew air to the outside. The fan pipe (**c**) was fitted on the top side and the mosquito release cup at bottom side of the release chamber (**d**). The mosquitoes that made a directional choice were trapped in either of the two trapping chambers (**e**) and data were recorded every morning by removing the fan pipe and the trapping chambers

Four two-port olfactometers were constructed from galvanized iron sheets (Fig. 1) to test the odour-orientation of gravid *An. gambiae* s.s. in response to test substrates. The olfactometers were placed in a netting-screened makeshift shed where experiments were run overnight under ambient conditions. The olfactometers had two large substrate holding chambers (1 × 0.9 × 1 m), two trapping chambers made of polyvinyl chloride (PVC) pipes (30 cm long and 10 cm diameter), a fan and mosquito release chamber (0.5 × 0.2 × 0.3 m). The size of substrate holding chambers was sufficient to carry whole live plants. Mosquitoes were introduced into the release chamber through an opening at the bottom. An electricity-powered fan drew air from the two substrate holding chambers through the holding chamber to the outside. Funnels inserted into the trapping chamber prevented mosquitoes from returning to the release chamber.

Test substrates were placed in both holding chambers. The fan was switched on five minutes before releasing 100 gravid *An. gambiae* s.s. to the choice chamber at 18:00. The choice made by mosquitoes was recorded the following morning at 8:00 by counting the number of mosquitoes trapped in each trapping chamber. The positions of the two test substrates were randomly rotated between chambers and olfactometers so that each substrate spent the same number of nights in each location.

All choice experiments are listed in Table 1. Prior to testing intact plants, the olfactometers were calibrated by evaluating their accuracy in generating valid and reproducible results and gauging the response rate that can be expected under standard test conditions. This was done by providing (1) two equal choices in both chambers (both containing water and both being empty) and (2) by providing two different choices with predictable outcomes (water vs empty; hay-infusion vs water).

**Table 1:** Summary of behavioural bioassays with gravid *An. gambiae* s.s. in two-port airflow olfactometers and in large field cages in relation to research questions

Treatment 1 (‘control’)	Treatment 2 (‘test’)	No. of replications	Total no. of gravid *An. gambiae* s.s. re-collected (out of total released)
Two-port airflow olfactometer bioassays			
Calibration experiments			
Do the olfactometer bioassays result in reproducible outcomes? What is the response rate that can be expected from released gravid mosquitoes?			
Water	Water	16	831 (1600)^a^
Empty	Empty	13	595 (1300)
Empty	Water	14	707 (1400)
Water	Hay infusion	12	710 (1200)
Choice between wet soil vs wet soil + graminoid plant from natural aquatic habitats			
Based on previous work on soil infusions [52], is the associated sedge, *C. rotundus,* attractive to gravid mosquitoes or is attraction based on soil alone?			
Water	Water	16	1060 (1600)
Soil	*C. rotundus*	16	875 (1600)
Choice between water vs water + graminoid plants			
Do intact graminoid plants from natural aquatic habitats attract gravid *An. gambiae* s.s.? Is *C. rotundus* more attractive than other graminoid plants? Is there a difference in behavioural response to a grass not naturally associated with breeding sites?			
Water	*C. rotundus*	16	1245 (1600)
Water	*C. exaltatus*	16	1204 (1600)
Water	*P. repens*	16	1194 (1600)
Water	*C. dactylon*	16	1016 (1600)
Water	*C. setaceus*	16	1064 (1600)
Choice between two graminoid plant species			
* P. repens*	*C. rotundus*	16	1224 (1600)
* C. dactylon*	*C. rotundus*	16	1179 (1600)
Large-cage choice bioassays with free-flying mosquitoes			
Do gravid *An. gambiae* s.s. show similar behavioural response to the plant volatiles at longer-range?			
Water	Water	16	1431 (3200)
Water	*C. rotundus*	16	2125 (3200)
Water	*C. exaltatus*	16	2075 (3200)
Water	*P. repens*	16	1858 (3200)
Water	*C. dactylon*	16	1988 (3200)
Water	*C. setaceus*	16	1478 (3200)
* P. repens*	*C. rotundus*	16	2234 (3200)

^a^Two-equal-choice bioassays using lake water were used as reference experiments. Modified BG-Sentinel mosquito traps were used in large-cage experiments

After calibration, a series of choice tests were done with intact plant materials (Table 1). Each comparison was replicated over 16 nights using a new batch of mosquitoes and fresh test substrates for every replicate. The replicate was discarded and repeated when mortality was ≥ 20% in the release/choice chamber or when less than 50% of the released mosquitoes responded (meaning majority remained in the central release chamber for the night).

### Large field-cage experiments with free-flying mosquitoes

**Fig. 2:**
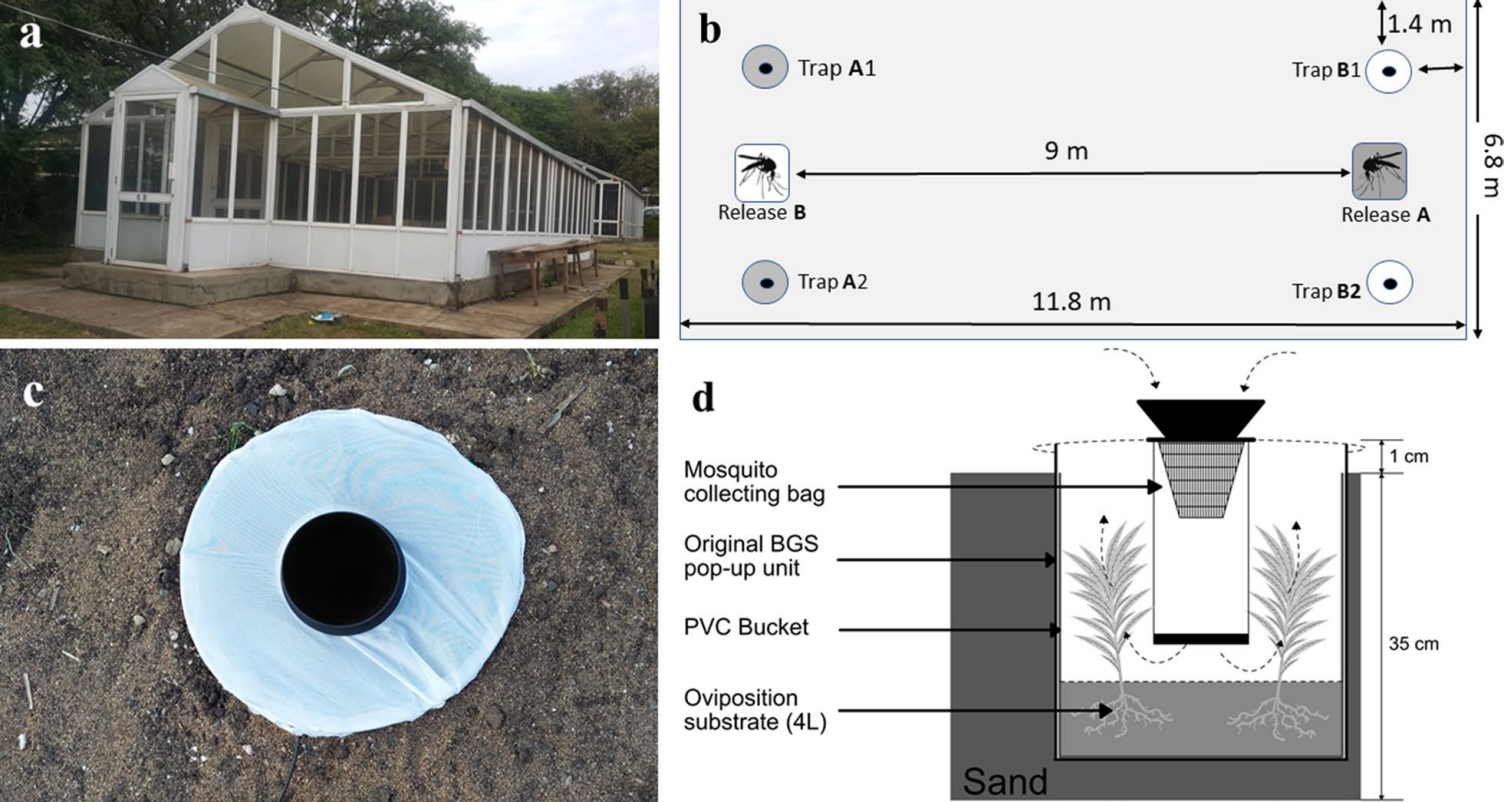
Overview of experimental set-up in the large field cages (**a**) with schematic overview of mosquito release points and trap positions (**b**). The white and blue colours show the trap locations and their respective mosquito release points. Test substrates were provided in modified BG-Sentinel traps buried in the ground (**c**). The cross-section through the modified BG-Sentinel gravid trap (**d**) shows the location of the plants and the airflow generated by the trap

Test treatments that elicited a positive response in olfactometer bioassays were then further evaluated with free-flying gravid *An. gambiae* s.s. in large field cages (11.8 m long × 6.8 m wide × 2.4 m high; Fig. 2A) under ambient environmental conditions to mimic a more natural setting and test for longer-range attraction. The test substrates were placed inside BG-Sentinel traps (Biogents AG, Regensburg, Germany) and these traps were buried in the ground so that only the netting top of the trap and collection funnel containing the fan were visible [51]. A black plastic bucket, 34 cm high and 30 cm in diameter, was inserted in each trap to hold the test substrates (Fig. 2B). Two traps with either equal or different test substrates included were set up per field cage (Table 1). The two traps were placed 4 m apart and 1.4 m away from the nearest wall. Mosquitoes were released from the opposite side, 9 m away from the traps (Fig. 2B). The two test substrates were allocated to the location randomly and the position of the two traps were exchanged between the two shorter walls of the cage in consecutive nights. Every experimental night, 200 gravid *An. gambiae* s.s. were released in the field cage at 18:00. The next morning at 08:00 the traps were collected, and the number of mosquitoes recaptured in the traps’ catch bags counted. Every experiment was repeated over 16 nights.

### Sample size considerations for bioassays

The sample size for replication was estimated using the formula developed by Hayes and Bennett [53] for comparing proportions of clustered data. For equal choices, an equal proportion responding to either choice was assumed for the reference (p1 = 0.5). Based on previous work [52], we aimed to be able to detect an increase in attraction by 16% (p2 = 0.66). Assuming a coefficient of variation (k) of 0.25 based on preliminary nightly test runs, and assuming at least 50 responding mosquitoes per night (n in each group), 16 replicates would be required for both treatment arms (p1 = equal choices; p2 = two choices) to detect the effect with 80% of power at a 5% significant level.

### Bioassay data analysis

The overall response rate of released mosquitoes was defined as the number of mosquitoes leaving the release chamber in either direction of the olfactometer; hence non-responders remained in the release chamber. Choice experiments using olfactometers and BG-sentinel traps were analysed with generalized linear models with quasi-binomial distributions fitted to cater for overdispersion. The proportions of gravid females responding to the ‘test’ (as opposed to the ‘control’) in two-choice experiments with two different choices were compared to the proportion of gravid mosquitoes responding to the ‘test’ in the experiments where ‘test’ and ‘control’ treatments were the same (lake water vs lake water) [54]. The experiment was included as the fixed factor and the ‘equal choice’ experiment was used as a reference to estimate the odds ratios (OR) and their 95% confidence intervals (CI). All reported mean proportions and their 95% confidence intervals (CIs) were estimated based on the model by transforming the log odds (logit) of the outcome to the odds scale and from the odds scale to the probability scale. R statistical software version 4.0.3 was used for the analyses [55].

### Sampling of headspace from intact plants

**Fig. 3:**
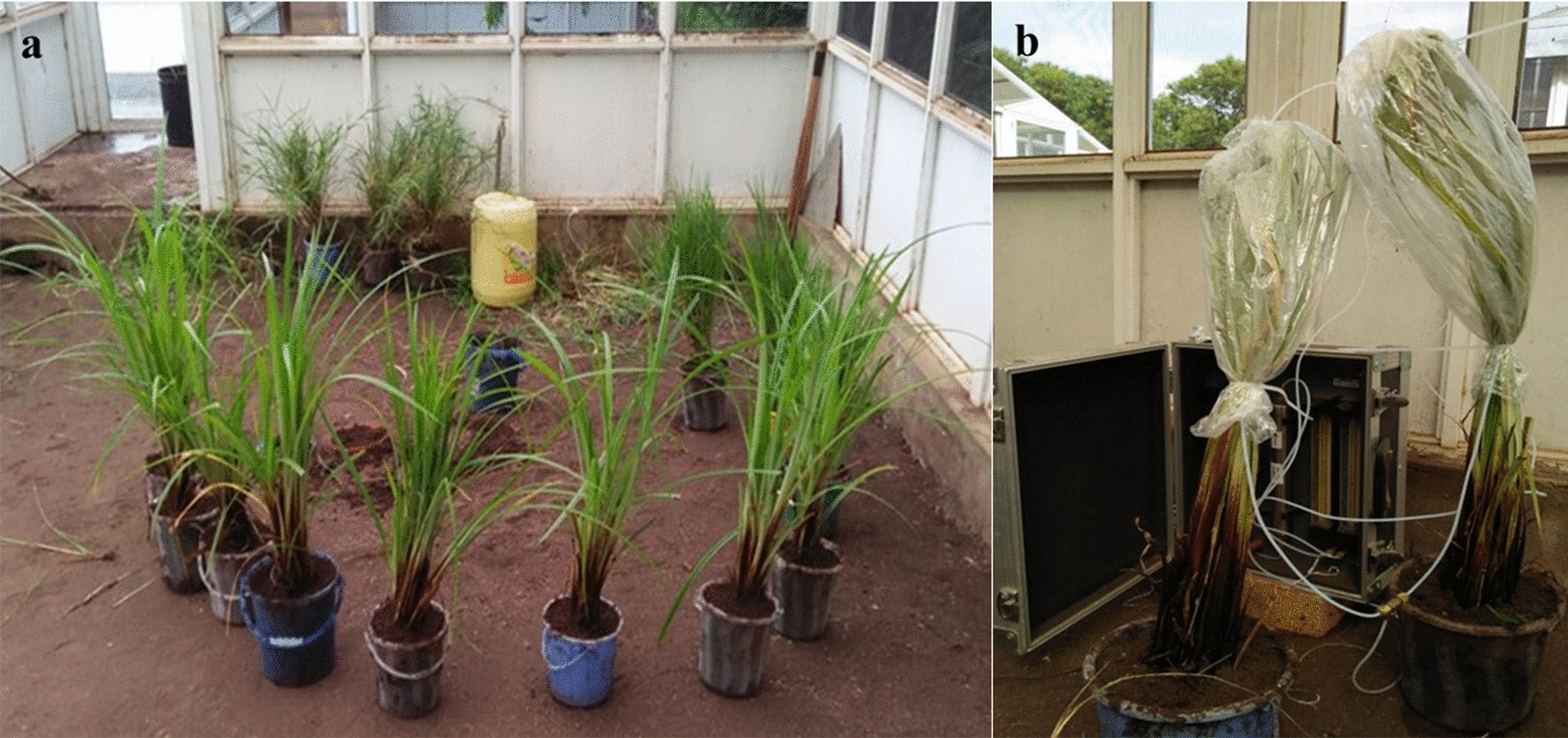
Plant preparation (**a**) for dynamic headspace sampling of volatile chemical compounds (**b**)

Volatile chemicals released from test plants were trapped from intact live plants using dynamic headspace (DHS) sampling. For this, several non-flowing plants (approximately 350 g) were placed with some soil in a bucket with water, similar to the experimental conditions. The sampling was done for 48 h under ambient conditions in the field cage (Fig. 3). The aerial parts of the intact plants were enclosed into heat-resistant roasting bags (Sainsbury’s Supermarkets Ltd, London EC1N 2HT) which were kept in an oven at 200 °C for 2 h prior to use. Porapak Q (50 mg, 50/80 mesh; Supelco) sorbent material was packed in a glass liner with glass wool on both ends to hold the sorbent in place. The Porapak Q traps were washed using 4 ml of hexane and kept in an oven for 2 h at 50 °C before use. Headspace collection was done by pumping 500 ml/min charcoal-filtered air into the bags through the inlet port and drawing the air out at a rate of 300 ml/min through the outlet port [56]. Headspace collections were done on two different dates, sampling four replicates of every plant species per date (total 8 headspace samples per plant species). Collections were also done from three replicates of empty cooking bags to account for the background chemicals concurrently for the two dates. After sampling, the traps were sealed with polytetrafluoroethylene (PTFE) tape and kept in a freezer at −71 °C. The filters were shipped to KTH Royal Institute of Technology, Stockholm, Sweden, where they were first eluted using 3 ml hexane to decrease the likelihood of chemicals remaining in the trap and then concentrated to 250 µl using a desiccator connected to a duo rotary vane pump before chemical analysis.

### Chemical analysis based on gas chromatography coupled with mass spectrometry

The headspace samples were analysed using a Trace 1300 gas-chromatograph (GC) coupled to an ISQ LT mass-spectrometer (MS; Thermo Fisher, Waltham, MA, USA). For each analysis, 1 μl of sample was injected in splitless mode. The temperature program started at 40 °C and was held for 1.8 min, after which the temperature was ramped to 200 °C at 20 °C/min. After reaching 200 °C, the ramp was changed to 50 °C/min until the temperature reached 240 °C, at which the temperature was held for 3 min. A 15 m × 0.25 mm × 0.25 µm (5% phenyl)-methylpolysiloxane column (Thermo Fisher) was used for all analyses. The carrier gas was helium and had a constant volumetric flow of 1 ml/min or a linear flow rate of 34 cm/s. The temperature of the transfer line between the GC and MS was set to 250 °C. The ionization source was an electron impact with ionization energy of 70 eV. Heptyl acetate was used as an internal standard to evaluate any instrumental variations for a selection of the replicate analyses. All GC–MS data was handled with Thermo Scientific™ Xcalibur™ software. Results from the mass spectrometry were submitted to the National Institute of Standards and Technology (NIST) MS Search 2.0 program for the NIST/US Environmental Protection Agency (EPA)/National Institutes of Health (NIH) Mass Spectral Library version 2.0g. The VOCs of the plants were identified using mass spectrometry (MS), retention time index (RI) and external standards (Ext Std). For each plant type, a minimum of two replicates from two different rounds were analysed to identify consistent compounds. For each plant type, one sample was also analysed three times to evaluate the variations in the same sample due to any possible instrumental drifts. For the calculation of the linear retention time index, the 49452-U C7-C40 alkane standard (Supelco, Bellefonte, PA, USA) was used as a reference. The cannabis terpene mix CRM 40755 (Sigma Aldrich, St. Louis, MO, USA) was used as external standards. The mix contained the following 20 terpenes α-pinene, β-pinene, camphene, 3-carene, α-terpinene, R-(+)-limonene, γ-terpinene, L-(−)-fenchone, fenchol, (1R)-(+)-camphor, isoborneol, menthol, citronellol, (+)-pulegone, geranyl acetate, α-cedrene, α-humulene, nerolidol, (+)-cedrol and α-(−)-bisabolol. This standard was complemented with the β-caryophyllene standard 22075 (Sigma-Aldrich) and the (–)-caryophyllene oxide 91034 (Sigma-Aldrich), to confirm the identified compounds. The area percentage was determined as the quotient between the area of compound peak as the numerator and the sum of all peaks detected in the corresponding chromatogram as the denominator. The mean area percentage was then calculated from all the DHS samples analysed and reported in the results. The peak areas were determined using the ICIS peak detection method in Xcalibur™ software.

## Results

### Two-port airflow olfactometer bioassays

The preliminary calibration experiments helped gauge the performance of the bioassay design and apparatus. During the majority of the preliminary experimental runs, around 50% of the released mosquitoes responded, whilst the others remained in the release chamber. This proportion could not be increased even when the experimental set up was modified. Hence, for all following experiments, it was defined that for a viable outcome the response rate must be 50% or above. When two equal choices of water were provided in the chambers, the released gravid mosquitoes distributed equally between the two chambers as expected (Table 2). When both chambers were empty, mosquitoes still responded, likely flying upwind in search of cues, and again distributed equally between the two chambers. The response rate, however, was overall slightly lower (46%) than when water was provided. When a choice between water in one chamber and no substrate in the other chamber was provided, > 80% of the responding females chose water. This confirmed that water vapour acts as an attractant for gravid mosquitoes. Moreover, it was confirmed that fermented 3-day-old hay infusion repels gravid *An. gambiae* s.s. Out of all responding females, > 70% oriented away from the infusion and towards the chamber with water.

**Table 2:** Preliminary olfactometer calibration experiments with gravid *An. gambiae* s.s.

Experiment	‘Control’ substrate	‘Test’ substrate	Percent (%) response of all released (95% CI)	Percent (%) attracted to ‘test’ of all responders (95% CI)
1	Empty	Empty	46 (38–53)	52 (46–58)
2	Empty	Lake water	51 (43–58)	80 (75–84)
3	Lake water	Lake water	52 (45–59)	49 (44–54)
4	Lake water	Infusion	59 (52–67)	29 (24–35)

*CI* confidence interval

**Fig. 4:**
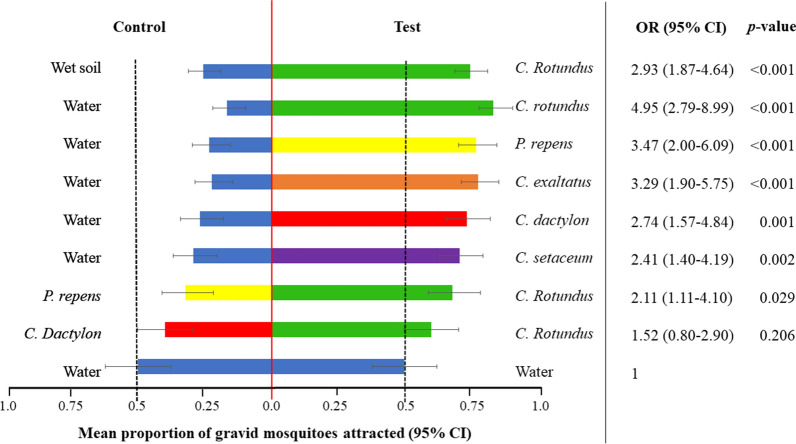
Short-range attraction of gravid *An. gambiae* s.s. to test substrates in choice experiments in two-port airflow olfactometers. The bars show the mean percentage with the 95% confidence intervals (CI). The outputs of the statistical analysis are presented as odds ratios (OR) and their 95% CI with the equal choice experiment as the reference. Each choice test was replicated over 16 different nights with 100 gravid *An. gambiae* s.s. released per replicate. Each substrate type is designated by a specific colour

After confirming the consistent performance of the bioassay, three sets of experiments were implemented. Equal choice experiment where the mosquitoes were provided with lake water in both chambers randomly allocated as ‘test’ and ‘control’, were set in parallel for all three sets of experiments. As expected, these reference test resulted in an approximate 1:1 distribution of gravid females (Fig. 4). Any preference for a specific test substrate in choice tests was expected to lead to a significant deviation from this balanced distribution.

Previous work [17] implicated soil from the *C. rotundus* collection site as attractive oviposition substrate for gravid *An. gambiae* s.s. Consequently, in a first step, we evaluated whether wet soil from the location might be equally or more attractive in olfactometer bioassays than the live *C. rotundus* plants in the same wet soil. However, the odds of a gravid female selecting the test chamber with the plants was nearly threefold higher than in the reference experiment (OR 2.93; Fig. 4). Removing the soil completely from the bioassay increased the odds further when compared to the reference (OR 4.95). Consequently, another four graminoid plants were tested and all of them released volatile chemicals attractive to gravid *An. gambiae* s.s. females (Fig. 4) in the airflow olfactometer. The odds of finding a gravid female in the test chamber with the plants were 2.4–5 times higher than in the reference experiment, with the most profound effect induced by *C. rotundus*. Even the drought-resistant *C. setaceus*, not naturally associated with mosquito breeding sites, elicited a significant positive orientation towards the plants’ odours (OR 2.41). The attractiveness of *C. rotundus* was further investigated when presented in choice tests with the Poaceae species, *P. repens* and *C. dactylon.* Chemical volatiles released from *C. rotundus* were preferred over the other grasses, though the effect size was moderate (Fig. 4).

### Large field-cage experiments with free-flying mosquitoes

**Fig. 5:**
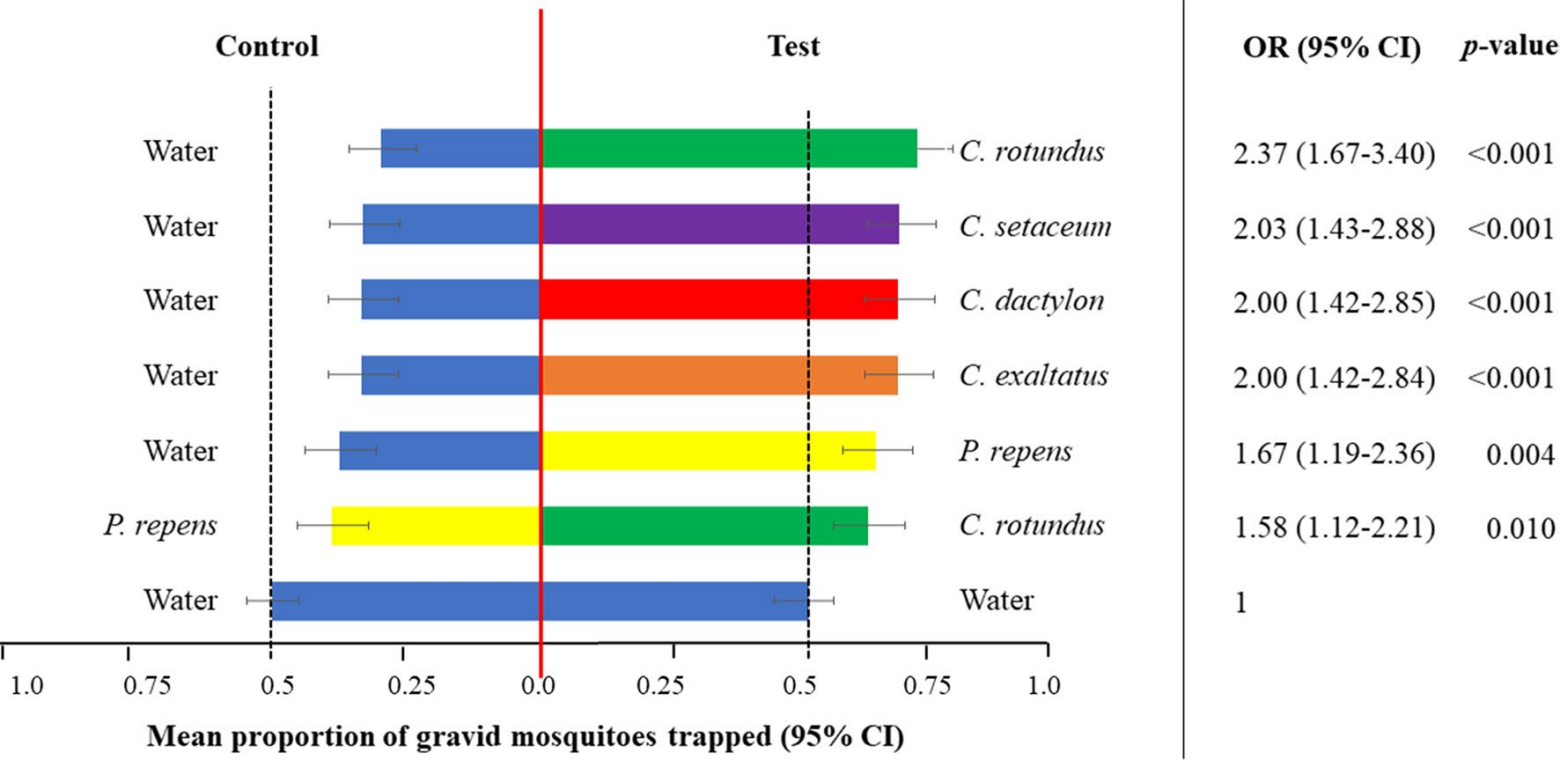
Long-range attraction of gravid *An. gambiae* s.s. to test substrates in choice experiments in large field cages. The bars show the mean percentage with the 95% confidence intervals (CI). The outputs of the statistical analysis are presented as odds ratios (OR) and their 95% CI with the equal choice experiment as the reference. Each choice test was replicated over 16 different nights with 200 gravid *An. gambiae* s.s. released per replicate. Each substrate type is designated by a specific colour

Bioassays with free-flying gravid mosquitoes confirmed olfactometer results with higher proportions of the released gravid females trapped with BG-Sentinel traps containing live plants than with traps that contained water only (Fig. 5). The odds of a female being captured in the test traps in the two-choice experiments were 1.5–2.5 times higher than in the reference experiment. Differences in the effect size of attraction between the plant species were not very pronounced under these more natural, long-range conditions, though *C. rotundus* volatiles did slightly outcompete volatiles from *P. repens* in a similar way as in the olfactometer bioassays (Fig. 5).

### Volatile organic compounds identified from the graminoid test plants

Chemical analyses were done for 21 headspace samples: *C. rotundus* (*n* = 5)*, C. dactylon* (*n* = 4), *C. exaltatus* (*n* = 4), *P. repens* (*n* = 4) and *C. setaceus* (*n* = 4). A total of 43 VOCs were detected with mass spectrometry (Table 3). A complete list of detected VOCs from each analysis of the different samples of the graminoid plants is shown in the Additional file 1: Table S1. The qualitative analysis shows that almost half of the detected compounds were sesquiterpenes. The second most common chemical class was monoterpenes, followed by a number of cyclic and straight compounds such as cyclic ketones, aliphatic esters and aromatic compounds. Table 3 shows compounds that have been detected in any one headspace sample of a plant species. There was a slight overlap in the profiles of monoterpenes and sesquiterpenes which were identified from different plant species (Table 3). Compounds such as limonene, β-caryophyllene, β-elemene, 1,1-dimethyl-3-methylene-2-vinylcyclohexane and α-guaiene were present in the headspace of at least three out of four graminoid plants. Unlike the other graminoid species, *C. setaceus*, contained more aromatic compounds and had less overlap with the other species in its chemical profile. Overall, roughly 10% of the VOCs were detected in the headspace of four of the five plants, while around 65% of the VOCs were detected from only a single species. This shows the diversity of the headspace in the chemical environment of the malaria vector.

**Table 3:** Volatile profile of dynamic headspace sampling of aerial parts from *C. rotundus* (CR), *C. exaltatus* (CE), *C. dactylon* (CD), *P. repens* (PR) and *C. setaceus* (CS)

Volatile compound	Area (%) composition ± SE						EAD Spec.	Physiol. stage	Ref.
	RI	CR	CE	CD	PR	CS			
Primary alcohol									
2-Ethyl-1-hexanol	1039	–	–	0.41 ± 0.322	–	–	–	–	
Aliphatic ketone									
Sulcatone	992	–	–	0.038a	–	–	Aa	G	[22]
Aliphatic ester									
4-Hexen-1-ol acetate	1012	–	–	3.338 ± 1.867	–	–	–	–	
Cycloalkane									
1-Isobutyl-1-cyclohexene	955	–	0.139a	–	–	–	–	–	
Cyclic ketone									
Cyclohexanone, 2,2,6-trimethyl	1043	–	–	0.102 ± 0.041	–	–	–	–	
Isophorone	1069	–	–	0.111 ± 0.088	–	–	–	–	
Aromatic									
1,4-Diethylbenzene	1056	–	–	–	–	1.433 ± 0.676	–	–	
Cymene	1062	–	–	–	–	0.351 ± 0.368	Aa, Ag	G	[22, 44, 57]
2,4-Dimethyl-acetophenone	1277	0.392 ± 0.324	–	–	–	0.514 ± 0.385	–	–	
β-Hydroxyethyl phenyl ether	1298	–	–	–	–	0.089a	–	–	
1H-indene, 1-ethylidene	1313	–	–	–	–	0.004a	–	–	
Alkyne									
4,6-Decadiyne	1063	–	–	–	–	0.475 ± 0.043	–	–	
Aromatic monoterpene									
Cumic alcohol	1271	0.418 ± 0.358	–	–	–	0.347 ± 0.308	–	–	
Monoterpene									
α-Pinene	942	–	–	0.035 ± 0.026	–	–	Aa	G	[22, 44]
β-Pinene	980	0.632 ± 0.287	0.021a	0.042 ± 0.035	–	–	Aa, Ag	G, U	[22, 57, 58]
Myrcene	994	0.452 ± 0.135	–	–	–	–	Ag	U	[59, 60]
Limonene	1035	2.805 ± 1.127	1.043 ± 0.31	0.088 ± 0.069	0.037 ± 0.018	–	Aa, Ag	G, U	[22, 43, 57, 58]
Eucalyptol	1039	–	–	–	0.877 ± 0.27	–	–	–	
4-Thujanol	1078	–	–	0.076 ± 0.067	–	–	–	–	
1,1-Dimethyl-3-methylene-2-vinylcyclohexane	1121	1.554 ± 0.672	0.78 ± 0.591	0.188a	–	0.072a	–	–	
Camphor	1158	–	–	0.028 ± 0.03	–	–	–	–	
β-Cyclocitral	1234	–	–	0.118 ± 0.069	0.025 ± 0.016	–	–	–	
Sesquiterpene									
Unidentified M = [204]*	1356	–	–	0.135 ± 0.06	–	–	–	–	
Ylangene	1362	–	–	–	0.115 ± 0.134	–	–	–	
Cyclosativene	1383	–	–	0.194 ± 0.081	–	–	–	–	
Copaene	1389	0.569 ± 0.372	–	0.044 ± 0.025	–	–	–	–	
γ-Elemene	1396	0.093 ± 0.028	–	–	–	–	Ag	U	[61]
β-Elemene	1404	3.64 ± 1.038	0.951a	0.069 ± 0.059	0.54 ± 0.19	–			
Cyperene	1418	0.584 ± 0.111	0.916 ± 0.514	–	–	–	–	–	
α-Gurjunene	1419	–	–	0.134 ± 0.113	–	–	–	–	
Cedrene	1436	–	0.101 ± 0.073	–	–	–	Ag	U	[59]
β-Caryophyllene	1438	3.517 ± 1.668	1.953 ± 0.641	0.141 ± 0.031	–	–	Aa, Ag	G, U	[22, 57, 59, 60]
α-Bergamotene	1448	–	–	0.096 ± 0.082	–	–	–	–	
β-Ionone	1453	–	–	0.115 ± 0.009	–	–	–	–	
Humulene	1473	2.376 ± 0.96	0.429 ± 0.279	–	–	–	Ag	U	[59, 60]
δ-Guaiene	1482	–	–	1.036 ± 1.696	–	–	–	–	
Germacrene D	1500	–	0.726 ± 0.471	0.126 ± 0.084	–	–	–	–	
α-Guaiene	1502	0.518 ± 0.2	0.132 ± 0.265	0.145 ± 0.163	0.072a	–	–	–	
α-Muurolene	1516	–	0.195 ± 0.109	0.099 ± 0.038	–	–	–	–	
δ-Cadinene	1535	–	0.796 ± 0.094	–	–	–	Ag	U	[59]
Caryophyllene oxide	1609	0.281 ± 0.165	–	–	–	–	–	–	
Humulene epoxide II	1639	0.591 ± 0.887	–	–	–	–	–	–	
Hexahydrofarnesyl acetone	1853	–	–	1.463 ± 0.379	–	–	–	–	

*RI* retention index calculated on a 15 m × 0.25 mm × 0.25 µm (5% phenyl)-methylpolysiloxane column. *SE* standard error. M = [204]* compound with the following 10 strongest MS peaks: 91(100), 105(98), 71(86), 133(77), 107(69), 93(62), 55(55), 77(52), 79(49), 69(47)

*a* No standard error is calculated, as the compound was only detected in one of the headspace samples. *Aa* *Anopheles arabiensis*; *Ag* *Anopheles gambiae* s.s. *EAD* electroantennogram detection published for *Anopheles* species. *G* EAD done for gravid females, *U* EAD done for unfed females

## Discussion

Our study confirms and expands the evidence that odour cues released from graminoid plants play a role in the orientation of gravid *An. gambiae* s.s. females. Volatiles released from these plants add significant attraction to water vapour alone. Generally, all graminoid plant species tested, including the dry-land ornamental grass *C. setaceus*, usually not associated with mosquito breeding sites, significantly attracted gravid females, and behavioural differences in response to different test plants were not very pronounced especially under the more natural, longer-range trapping conditions.

Whilst the behavioural response of gravid *An. gambiae* s.s. mosquitoes appeared to be slightly stronger in reaction to the sedge, *C. rotundus,* than to most other test plants, we were not able to exactly establish any unique differences in the chemical profiles that might explain this. This is likely, in part, due to the chemical sampling method. To the best of our knowledge, our bioassays are the first to use live plants rather than eluted headspace extracts for testing for attractiveness to gravid malaria vectors. Our aim was to test the behavioural response of gravid females to plant volatiles under as natural conditions as possible. Plant volatiles react differentially with atmospheric oxidants, such as ozone, resulting in odour plumes that not only include the plant-emitted volatile chemicals but also gradually include a blend of degradation products [36], which might not be picked up during DHS sampling with filtered air. We had opted for headspace sampling, since it is a non-destructive method for sampling the volatile profile emitted by plants which might consequently be detected by insects [62]. The pooled analyses of our headspace samples suggest that there are variations between the chemical profiles of the different plant species. It is unclear, however, whether these differences would be consistent over time and under different environmental conditions, and whether they are responsible for the variations observed in attracting gravid females in the bioassays. Our GC results have been highly variable between replicate plant samples of the same species (Additional file 1: Table S1) with some samples not resulting in any detectable compounds. This is not unexpected, given that we have taken only a ‘snapshot’ of volatiles released at a particular time point and without carefully standardizing plant age and development. Some volatiles may be emitted in quantities below technical detectability, yet these might be functionally relevant for insect attraction [36]. Volatile organic chemicals emissions and concentrations are also affected by light, temperature, nutritional and soil-moisture conditions, and even by species composition of the neighbouring plant community [63–69]. Abiotic stresses, including stress induced by the air sampling itself when plant material is enclosed in plastic bags will also affect the volatile profile. Going forward, it will be desirable to sample under natural, yet varying environmental conditions and to compare results across different sampling strategies [62] for a better understanding of the composition and concentration of compounds in the headspace of plants that might affect natural mosquito behaviour.

In our study, and across published work, we see very little variation in the strengths of the behavioural response of gravid mosquitoes to varied graminoid plant species, despite the fact that volatile profiles appear variable. The behavioural response of gravid *An. gambiae* s.s. induced by the wild graminoid plants in our bioassays was in the same ranges as those reported previously for *An. arabiensis* and *An. coluzzi* in response to low release rates of headspace extracts from rice plants [22] and from the tropical African wetland grasses (Poaceae) *Echinochloa pyramidalis, E. stagnina* and *Typha latifolia* [42]. It was also in a similar range as observed for the attraction of unfed females to plant-based volatiles [58, 60, 70]. A limitation of our study was our inability to access equipment for electroantennography to determine exactly which volatile chemicals released from the test plants were detected by the gravid female’s antenna. However, when comparing the volatile chemicals identified in our study with those published for rice plants and pollen from sugar cane and maize in the context of oviposition [22, 43, 44], as well as with those published for a range of plants preferentially visited by malaria vectors for sugar feeding [32, 58–60], it becomes apparent that there is significant overlap in the chemical compositions. Compounds reported here, such as limonene, α- and β-pinene, p-cymene, sulcatone, humulene, cedrene, β-myrcene, and β-caryophyllene, have previously been reported to elicit electrophysiological responses in gravid and unfed female *Anopheles* [22, 43, 44, 57–60], and many of them have been formulated into synthetic blends and shown to be attractive to unfed and gravid *Anopheles* under highly standardized experimental conditions [22, 43, 58, 71]. These compounds are among the most common VOCs emitted from plants [72] since they are synthesized through biosynthetic pathways common in most plants [39, 73, 74].

In our study, three volatile chemicals, namely 1,1-dimethyl-3-methylene-2-vinylcyclohexane, α-guaiene and β-elemene, have not been tested previously, yet were detected frequently in four out of the five test plants. It might be useful to explore their potential to manipulate odour-orientation of *Anopheles* mosquitoes in follow-up studies, since they have been implicated as semiochemicals for other insect species [75–79]. For example, 1,1-dimethyl-3-methylene-2-vinylcyclohexane was attractive to the beech leaf-mining weevil [76], guaiene has been suggested to play a role in the attraction of the litchi stem-end borer [80] and β-elemene has been implied to contribute to attraction of the gravid tobacco moths [77] and the white-spotted longhorn beetle [78].

Myrcene, γ-elemene, humulene epoxide II and hexahydrofarnesyl acetone were specific to headspace samples of *C. rotundus* in our analysis. This does not, however, necessarily imply that these compounds contributed to the attractiveness in our bioassays. Information on these compounds as info-chemicals for insects and specifically mosquitoes is scant and none of them have been tested with gravid malaria vectors. Both unfed *Anopheles* and unfed *Aedes* mosquitoes showed electrophysiological activity to β-myrcene in previous studies [60, 70]. It was observed that myrcene elicits an avoidance behaviour in unfed *An. gambiae* searching for sugar [60] or blood meals [81]. γ-Elemene was identified from plant headspace and found to be electrophysiologically active for unfed *An. gambiae*, but behavioural implications were not studied [61].

Gravid malaria vectors navigate a complex chemical environment in search of oviposition sites. It is plausible to assume that volatile chemical cues emanating from aquatic habitats and their surroundings are only used at relatively short-range, with visual cues and air movements guiding the gravid females’ flight towards a water body [1, 82]. Visual cues will include near-infrared radiation from slowly released heat from water bodies in the evening [83], polarized light from water surfaces [84], as well as ultraviolet light [85], all of which present strong long-range cues likely used by gravid mosquitoes to evaluate the location and quality of potential oviposition sites [2]. In this context, therefore, it remains unclear whether attractive, yet common, plant-based semiochemicals in odour-baited traps will be able to compete in an attract-and-kill approach, with the complex interaction of cues provided by natural aquatic habitats. To date over 100 semiochemicals have been identified for mosquitoes of all physiological stages, yet synthetic odour-baited traps hardly play any role in contemporary surveillance and control of malaria vector mosquitoes [4]. Synthetic odour-baits mimicking human body odour have been shown to perform poorly in attracting host-seeking *Anopheles* mosquitoes when present in close proximity to natural human blood hosts [86] and field evaluations of the oviposition attractant cedrol, showed that visual cues provided by an open water surface were essential in combination with the chemical cue to attract wild oviposition-site searching females [17]. In order to develop vector control interventions that manipulate the odour-orientation of malaria vectors in their natural environment, less emphasis might be placed in future on detecting more semiochemicals but more emphasis on how to formulate and present these chemicals in combination with other essential cues used by mosquitoes, to improve the efficacy of such interventions [4].

## Conclusions

Our results suggest that plant volatiles provide a more general cue for gravid malaria vectors rather than vectors being highly adapted and evolved in context to specific plant species and environments. All the graminoid test plants were very common, occurring in high abundance in grasslands and wetlands in sub-Saharan Africa and beyond [87–91]. Our results also challenge a previous suggestion [42] that volatile chemicals released from the grass family Poaceae are in general more attractive to gravid *Anopheles* mosquitoes than those released from the sedge family Cyperaceae. The variations in chemical profiles and behavioural responses have been shown to be subtle across all studies. Productive breeding sites have been associated with species from both plant families in a number of field surveys [24, 26, 29, 40]. In nature, plant-based chemical cues interact with many other biotic and abiotic environmental cues to help gravid malaria vectors to orient and select suitable egg-laying sites, including non-plant-based chemicals [17, 18, 41, 92, 93], light and reflection [82], contrast [94], structure including plant height [29], conspecific immature stages [16, 95, 96], and other macroinvertebrates [14, 97]. These complex interactions will need to be taken into consideration when designing ‘attract-and-kill’ strategies targeting gravid vectors with odour-baited traps.

## Supplementary Information

**Additional file 1.** A complete list of detected volatile organic compounds from each analysis of the different samples of *Cyperus rotundus* (CR), *Cyperus exaltatus* (CE), *Cynodon dactylon* (CD), *Panicum repens* (PR) and *Cenchrus setaceum* (CS).

## Data Availability

The datasets for the plant headspace chemical analysis can be found at Figshare https://doi.org/10.6084/m9.figshare.14790162. The primary datasets generated from the bioassays and analysed are available from the corresponding authors on reasonable request.

## Abbreviations

GC–MS: Gas chromatography-mass spectrometry
*icipe*-TOC: International Centre of Insect Physiology and Ecology–Thomas Odhiambo Campus
PVC: Polyvinyl chloride
LLIN: Long-lasting insecticide nets
IRS: Indoor residual sprays
VOC: Volatile organic compound
WHO: World Health Organization
